# Incidence and risk factors of joint stiffness after Anterior Cruciate Ligament reconstruction

**DOI:** 10.1186/s13018-020-01694-7

**Published:** 2020-05-14

**Authors:** Bin Wang, Jun-Long Zhong, Xiang-He Xu, Jie Shang, Nan Lin, Hua-Ding Lu

**Affiliations:** Department of Orthopaedics, The Fifth Affiliated Hospital of Sun Yat-Sen University, Zhuhai, 519000 Guangdong China

**Keywords:** Joint stiffness, Anterior cruciate ligament reconstruction, Meta-analysis, Incidence, Risk factors

## Abstract

**Background:**

Joint stiffness is a common complication after anterior cruciate ligament (ACL) reconstruction, which seriously affects the efficacy of the operation and patient satisfaction. After ACL reconstruction, the identification of joint stiffness’ risk factors can help its prevention. This meta-analysis was conducted to evaluate joint stiffness’ risk factors and incidence after ACL reconstruction and provide guidance on its prevention.

**Methods:**

PubMed, Embase, and Cochrane Library were searched to obtain relevant studies. The odds ratios (ORs) with 95% confidence intervals (CIs) for all potential risk factors were analyzed using fixed or random-effects meta-analysis in RevMan 5.2.

**Results:**

In total, there were 37 studies and 113,740 patients that were included in this study. After ACL reconstruction, joint stiffness’ incidence negatively correlated with the studies publication time (*R* = −0.62, *P* = 0.0094). After ACL reconstruction, the joint stiffness overall pooled incidence was 3% (95% CI, 3-4%). Gender (OR, 0.51; 95% CI, 0.38-0.68; *P* < 0.00001) was identified as a risk factor. Potential risk factors, such as trauma to surgery time interval, graft type, and concomitant surgery with meniscus injury, have no significant correlation with joint stiffness after ACL reconstruction.

**Conclusion:**

This study indicated that joint stiffness’ incidence after ACL reconstruction is 3% and that gender is a risk factor for joint stiffness after ACL reconstruction.

## Background

Anterior cruciate ligament (ACL) injuries account for a large proportion of knee injuries and have a significant impact on knee joint stability [1]. With the development of sports’ medicine, arthroscopic ACL reconstruction has proven to be a safe and effective surgical method [2, 3]. Nonetheless, knee stiffness, a common postoperative complication, severely restricts patients from returning to their original exercise level [4]. Knee postoperative stiffness manifests as an insufficient range of motion, which can be caused by poor graft position, cyclops lesions, and arthrofibrosis [5–7]. Previous studies reported that after ACL reconstruction, the incidence of joint stiffness was between 4 and 38% [8].

Due to the effect of joint stiffness on efficacy and patient satisfaction following ACL reconstruction, the identification and minimization of risk factors’ occurrence, are essential. Sanders et al. [9] reported that female joint stiffness’ incidence was significantly higher than that in men; however in another report, it was shown that womanhood is not a risk factor for joint stiffness [10]. Controversies also exist with regard to the time interval from trauma to surgery, the type of graft and concomitant surgery with meniscus injury [11–15].

Therefore, we conducted this meta-analysis to investigate joint stiffness risk factors and incidence after ACL reconstruction and provide guidance on the joint stiffness’ prevention to improve ACL reconstruction efficacy and post-operative patients’ satisfaction.

## Methods

### Search strategy

The systematic review and meta-analysis methods used in this study followed the recommendations of Moher et al [16]. Using the databases of Cochrane Library, PubMed, and Embase, a systematic literature search was performed for studies on joint stiffness in patients after ACL reconstruction on February 18, 2019. The retrieval strategy used the following terms in the title and abstract: (“anterior cruciate ligament” OR “ACL”) AND (“reconstruction” OR “treatment” OR “surgery” OR “repair”) AND (“stiffness” OR “range of motion deficits” OR “ROM deficits” OR “arthrofibrosis”).

### Eligibility criteria

Studies that met the following inclusion criteria were included in our meta-analysis:

1. The studies should be randomized or non-randomized controlled studies or observational studies.
2. The studies should contain sufficient information on joint stiffness risk factors and incidence after ACL reconstruction.
3. The object of the study must be human participants.
4. The language of the article must be English or Chinese.

Studies that met the following exclusion criteria were removed from our meta-analysis:

1. Conference abstracts, letters, editorials, case reports, and reviews.
2. Joint stiffness was not present in the clinical results of all study participants.
3. Insufficient control information in the study which limits complete extraction.

### Data extraction

The following information was independently extracted by the two authors (WB and ZJL) using a standardized Excel table: (1) The baseline characteristics of the included literature comprised representative authors, publication time, nationality, study type, study period, number of included patients, time and number of patients who were followed up, and number of patients with joint stiffness and joint stiffness incidence; (2) Related risk factors mentioned in three or more studies.

### Quality assessment

We evaluated the quality of included studies using the Newcastle-Ottawa quality assessment scale [17]. Studies with a quality of more than five stars were included in future analyses.

### Statistical analysis

Joint stiffness incidence after ACL reconstruction was determined using inverse variance in statistical methods and risk difference in effect, measured with 95% confidence intervals (CIs). The binary variables of potential risk factors were performed using Mantel-Haenszel in statistical methods and odds ratio in effect, measured with 95% CIs. To identify the heterogeneity of the included studies, we performed a chi-square test and calculation of *I*^2^ statistics. We considered *I*^2^ ≤ 50% and/or *P* ≥ 0.1 to be an insignificant heterogeneity. In the above heterogeneous outcome, we applied the fixed effect model in the analytic model for statistical processing. On the contrary, we used the random effect model. The above statistical analyses were performed using the Review Manager 5.2. The R software was used to fit the correlation between incidence and the studies’ publication time using Spearman analysis. *P* < 0.05 was considered statistically significant.

## Results

### Study selection and characteristics

**Fig. 1:**
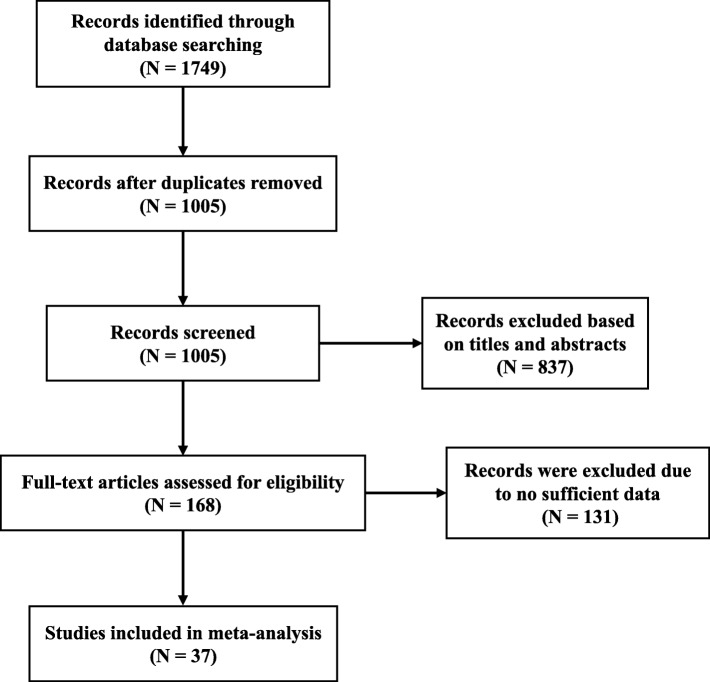
Preferred reporting items for systematic meta-analyses (PRISMA) flow diagram of the study selection process

Using the pre-designed search strategy, we identified a total of 1749 records from three databases. After removing duplicate results, 1005 potential results were screened for the follow-up study and via intensive reading of the article title and abstract; we further identified 168 studies to be included in the follow-up research process. Next, we downloaded and carefully screened the full text of the selected articles. As a result, 131 articles were excluded due to insufficient data identification. Finally, 37 studies were included in this meta-analysis and a detailed screening process was recorded in a flow diagram (Fig. 1). The included studies’ baseline characteristics were detailed in Table 1.

**Table 1:** Characteristics of included studies

Patients, *n*		Stiffness		
Included	Followed up	Time of follow-up	Total number of stiffness, *n*	Incidence of stiffness (%)
64	32	5 years	23	71
959	959	9 months	42	4
106	87	18 months	13	15
194	194	Minimum 12 months	38	19.6
57	57	Minimum 33 months	8	14
191	188	Average 16 months (3-60 months)	22	11.7
37	37	Minimum 6 weeks	1	2.7
162	105	Average 40 months (24-76 months)	2	1.9
31	31	52 weeks	2	6.5
19	19	Minimum 2 years	1	5.3
49	49	Minimum 6 months	3	6.1
133	120	Average 54.4 months (24-104 months)	1	0.8
100	100	Minimum 12 months	12	12
1016	933	Average 6.3 years (1.6-14.2 years)	77	8.3
40	40	Average 24.3 months (21-28 months)	15	37.5
75	66	Average 22 months (16-29 months)	5	7.6
980	920	Minimum 2 years	40	4.3
14522	14522	Average 1.9 years	95	0.7
103	103	Average 21 months (6-66 months)	2	1.9
59244	59244	Minimum 3 months	955	1.6
13358	13358	Minimum 3 months	298	2.2
1841	1355	Average 10.3 years	23	1.7
1205	1112	2 years	16	1.4
26	26	Minimum 12 months	6	23
969	969	Minimum 45 days	1	0.1
102	73	Minimum 3 months	6	8.2
1121	1121	Minimum 3 months	20	1.8
2558	2424	Average 56.7 months (7.6-124 months)	108	4.5
59	57	Minimum 3 months	14	24.6
27	27	2 years	4	14.8
127	127	Average 10.1 months	19	15
9766	9766	Average 9.2 weeks	111	1.14
424	424	37 months	38	9
358	358	Minimum 3.5 months	10	2.8
958	811	24 months	72	8.8
200	166	6 months	8	5
2559	2559	3 months	6	0.23

### Quality assessment of the studies

According to the Newcastle-Ottawa quality assessment scale, we have quantified the quality of the included studies, and the results’ details are presented in Table 2. The quality of the included studies was acceptable as there were 24 studies with eight stars and 13 articles with seven stars.

**Table 2:** Quality assessment of included studies

Study	Selection	Comparability	Exposure/outcome	Total
Feagin et al. [18]	3	2	3	8
Fisher et al. [19]	2	2	3	7
Wasilewski et al. [20]	2	2	3	7
Dandy et al. [21]	2	2	3	7
Wachtl et al. [22]	2	2	3	7
Cosgarea et al. [23]	3	2	3	8
Kao et al. [24]	2	2	3	7
Orfaly et al. [25]	3	2	2	7
Meighan et al. [26]	3	2	3	8
Millett et al. [27]	2	2	3	7
Nicholas et al. [28]	2	2	3	7
Prodromos et al. [29]	3	2	3	8
Robertson et al. [8]	3	2	3	8
Nwachukwu et al. [6]	3	2	3	8
Demirağ et al. [30]	3	2	3	8
Kiekara et al. [31]	3	2	3	8
Hettrich et al. [32]	3	2	3	8
Csintalan et al. [33]	3	2	3	8
Cruz et al. [34]	3	2	3	8
Werner et al. [35]	3	2	3	8
Cancienne et al. [36]	3	2	3	8
Sanders et al. [9]	3	2	2	7
Ding et al. [37]	3	2	3	8
Meister et al. [38]	2	2	3	7
Bordes et al. [39]	3	2	2	7
Runner et al. [40]	3	2	3	8
Su et al. [41]	3	2	3	8
Huleatt et al. [12]	3	2	3	8
Osti et al. [42]	3	2	3	8
Westermann et al. [43]	3	2	3	8
Patel et al. [44]	3	2	3	8
Cruz et al. [45]	3	2	2	7
Offerhaus et al. [46]	3	2	3	8
Panisset et al. [47]	2	2	3	7
Romain et al., 2019	3	2	3	8
Rushdi et al. [15]	3	2	3	8
Grassi et al. [48]	3	2	3	8

### Incidence

**Fig. 2:**
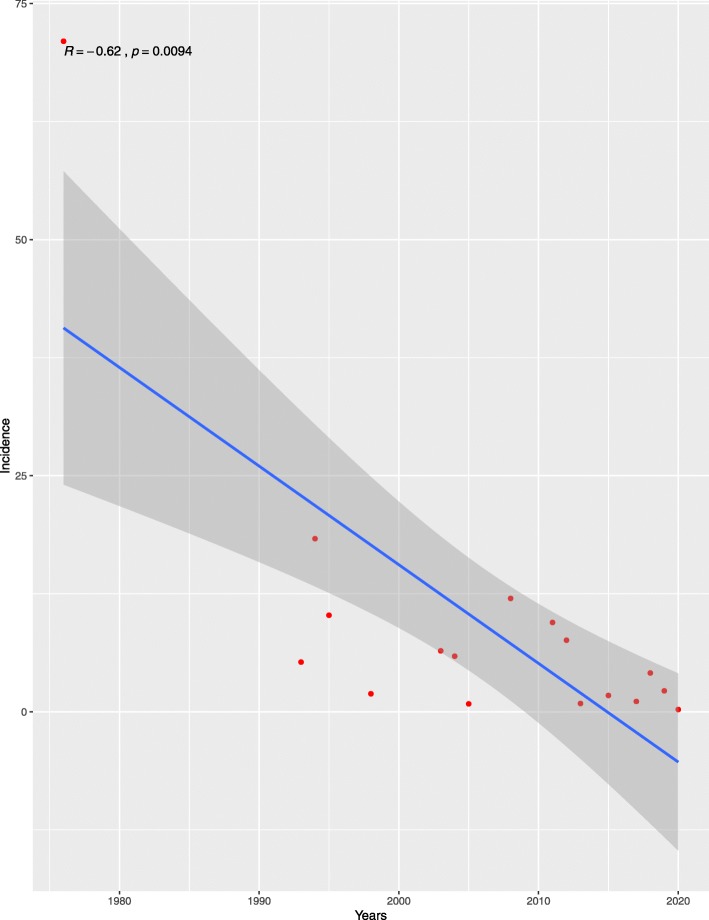
Correlation between the incidence of joint stiffness after ACL reconstruction and the studies publication time

**Fig. 3:**
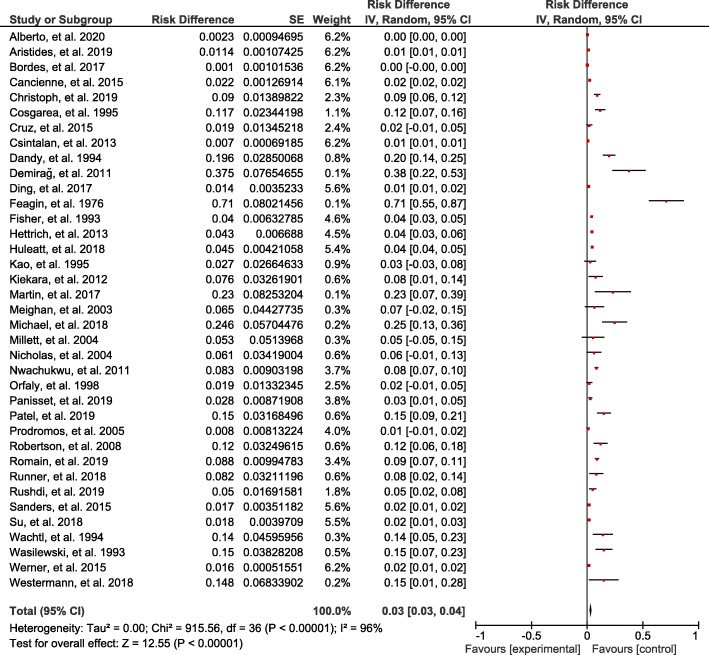
Joint stiffness’ pooled incidence after ACL reconstruction. IV, inverse variance; CI, confidence interval

In total, there were 37 studies and 113,740 patients that were included in this study. The results showed that 2117 patients encountered joint stiffness after ACL reconstruction and the reported incidence rates by various institutes ranged from 0.1 to 71%, showing large fluctuations. After ACL reconstruction, the joint stiffness’ incidence negatively correlated with the studies’ publication time (*R* = −0.62, *p* = 0.0094) (Fig. 2). After ACL reconstruction, the overall pooled incidence of joint stiffness was 3% (95% CI, 3-4%) (Fig. 3).

### Risk factors for joint stiffness after ACL reconstruction

#### Gender

**Fig. 4:**
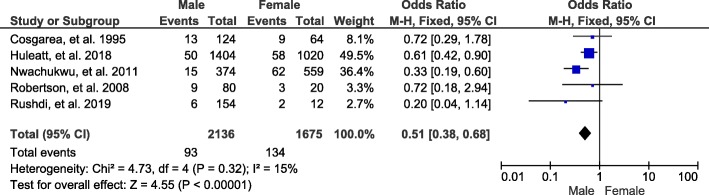
Forest plot of joint stiffness between male and female groups after ACL reconstruction. M-H, Mantel-Haenszel; CI, confidence interval

A total of 5 studies and 3811 patients were included in this study group, and the results showed that gender is a risk factor for joint stiffness after ACL reconstruction (OR, 0.51; 95% CI, 0.38-0.68; *p* < 0.00001) (Fig. 4).

#### Time interval from trauma to surgery

**Fig. 5:**
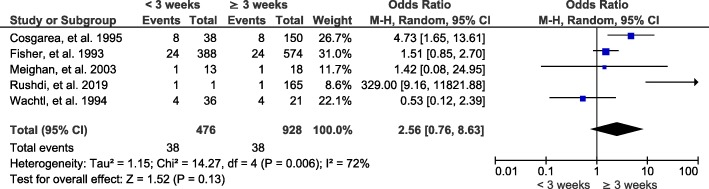
Forest plot of joint stiffness after ACL reconstruction between the time interval from trauma to surgery for less than 3 weeks and for more than 3 weeks (group 2). M-H, Mantel-Haenszel; CI, confidence interval

A total of 5 studies and 1404 patients were included in this study group, and the results showed that there is no significant correlation between the time interval from trauma to surgery and joint stiffness after ACL reconstruction (OR, 2.56; 95% CI, 0.76-8.63; *P* = 0.13) (Fig. 5).

#### Graft type

**Fig. 6:**
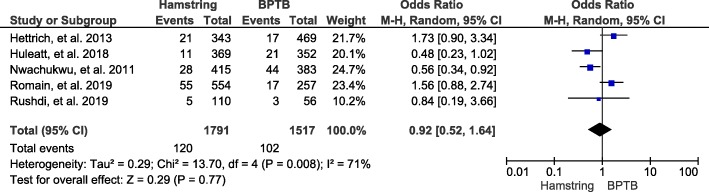
Forest plot of joint stiffness between hamstring and BPTB groups after ACL reconstruction. BPTB, bone-patellar tendon-bone; M-H, Mantel-Haenszel; CI, confidence interval

A total of 5 studies and 3308 patients were included in this study group, and the results showed that there is no significant correlation between the type of graft and joint stiffness after ACL reconstruction (OR, 0.92; 95% CI, 0.52-1.64; *P* = 0.77) (Fig. 6).

#### Concomitant surgery with meniscus injury

**Fig. 7:**
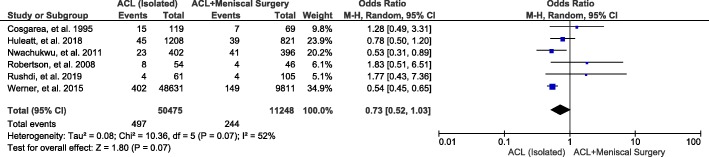
Forest plot of joint stiffness between the ACL (isolated) and ACL + meniscal surgery groups after ACL reconstruction. M-H, Mantel-Haenszel; CI, confidence interval

A total of 6 studies and 61,723 patients were included in this study group, and the results showed that there is no significant correlation between concomitant surgery with meniscus injury and joint stiffness after ACL reconstruction (OR, 0.73; 95% CI, 0.52-1.03; *P* = 0.07) (Fig. 7).

## Discussion

In this study, we found that the incidence of joint stiffness after ACL reconstruction varies from 0.1 to 71% with a relatively large fluctuation amplitude [6, 8, 9, 14, 15, 18–49]. After statistical analysis, we observed that the incidence was negatively related to the study publication time. Retrospectively, we found that ACL knowledge and research began in the mid-nineteenth century, and it was not until the early twentieth century that there was a proposal for ACL reconstruction [50]. With the advances in ACL anatomy and biomechanical research, the improvement of ACL injury diagnosis, the development of ACL surgery technology, and the concept of rehabilitation, the postoperative complications of ACL reconstruction, including joint stiffness, were significantly reduced and the curative effect significantly improved [51, 52]. However, once joint stiffness occurs, it can have a significant impact on patients’ quality of life and may require secondary surgery [53]. To avoid joint stiffness, particular attention to the related risk factors is required to pay attention.

The pooled results indicated that gender was a risk factor for joint stiffness after ACL reconstruction. When ignoring other related risk factors, the incidence of joint stiffness was significantly higher in women than that in men. Previous studies have reported that the female athletes’ risk of ACL injury is 2 to 6 times higher than that in male athletes [54]. The structural difference between male and female athletes can be used as an anatomical factor to explain the above phenomenon [55]. It was also shown that ACL injury occurs more frequently in women pre-ovulation stage, which is related to the effects of estrogen, progesterone, testosterone, and relaxin on women’s ligaments [56, 57]. Park et al. reported that knee joint laxity and stiffness’ change is related to ovulation hormone levels [58]. Given that women are a common risk factor for ACL injury and postoperative joint stiffness, more attention should be paid to this factor by fully evaluating the patient’s hormone levels, choosing the appropriate timing of surgery and improving the efficacy of surgery.

Our pooled results showed that the time interval from trauma to surgery has no significant correlation with joint stiffness after ACL reconstruction. Our results were consistent with previous reports that indicated that early ACL reconstruction surgery, within 3 weeks or even 1 week after trauma, does not increase the risk of postoperative joint stiffness [11, 13]. The most commonly used autografts for ACL reconstruction are the hamstring and the bone-patellar tendon-bone [59]. Despite their advantages and disadvantages, failure rates are low and there is no difference in graft fracture [60, 61]. Our results showed that there was no significant correlation between these two autografts and joint stiffness; therefore, both types of grafts can be used for ACL reconstruction, and the choice depends on the patient individual specificity. According to the literature, meniscal injury is associated with 40% to 60% of patients with ACL injury [62]. Meniscus plays very important roles in knee joints stability, stress transmission, proprioception, and joints’ lubrication and nutrition [63]. Many scholars have shown that the outcomes of ACL reconstruction alone, or in combination with a meniscus operation, are similar [64]. Our analysis also showed that simultaneous meniscus related surgery did not increase the risk of joint stiffness. Due to the important functions of the meniscus, we should select the appropriate treatment method according to the condition of the meniscal injury and its complete treatment.

Some limitations existed in this meta-analysis. First, most of the included studies were retrospective, which may have affected the results’ credibility. Second, there is a clinical heterogeneity that cannot be eliminated through subgroup analysis, which may be caused by differences in patients’ standards, included in each study, and the surgeons’ surgical techniques. In addition, there were some potential risk factors, such as age, weight, rehabilitation training, and preoperative activity limitation, which were not included in our analysis due to insufficient data. Despite these limitations, we believe that this study deepens our understanding of joint stiffness and provides guidance for preventing joint stiffness after ACL reconstruction. In the future, further studies will be needed to investigate the risk factors of joint stiffness after ACL reconstruction.

## Conclusion

This study indicated that the incidence of joint stiffness after ACL reconstruction is 3%. Gender is a risk factor for joint stiffness after ACL reconstruction.

## Data Availability

The datasets used and/or analyzed during the current study are available from the corresponding author on reasonable request.

## Abbreviations

ACL: Anterior cruciate ligament
OR: Odds ratios
CI: Confidence intervals
